# Review: Mechanisms and perspective treatment of radioresistance in non-small cell lung cancer

**DOI:** 10.3389/fimmu.2023.1133899

**Published:** 2023-02-14

**Authors:** Ting Zhou, Li-Ying Zhang, Jian-Zheng He, Zhi-Ming Miao, Yang-Yang Li, Yi-Ming Zhang, Zhi-Wei Liu, Shang-Zu Zhang, Yan Chen, Gu-Cheng Zhou, Yong-Qi Liu

**Affiliations:** ^1^ Provincial-Level Key Laboratory for Molecular Medicine of Major Diseases and The Prevention and Treatment with Traditional Chinese Medicine Research in Gansu Colleges and University, Gansu University of Chinese Medicine, Lanzhou, China; ^2^ Experimental & Training Teaching Centers, Gansu University of Chinese Medicine, Lanzhou, China; ^3^ College of Basic Medicine, Gansu University of Chinese Medicine, Lanzhou, China; ^4^ Key Laboratory of Dunhuang Medicine and Transformation at Provincial and Ministerial Level, Gansu University of Chinese Medicine, Lanzhou, China

**Keywords:** non-small cell lung cancer, radiotherapy, radioresistance mechanisms, toxicity, Traditional Chinese Medicine

## Abstract

Radiotherapy is the major treatment of non-small cell lung cancer (NSCLC). The radioresistance and toxicity are the main obstacles that leading to therapeutic failure and poor prognosis. Oncogenic mutation, cancer stem cells (CSCs), tumor hypoxia, DNA damage repair, epithelial-mesenchymal transition (EMT), and tumor microenvironment (TME) may dominate the occurrence of radioresistance at different stages of radiotherapy. Chemotherapy drugs, targeted drugs, and immune checkpoint inhibitors are combined with radiotherapy to treat NSCLC to improve the efficacy. This article reviews the potential mechanism of radioresistance in NSCLC, and discusses the current drug research to overcome radioresistance and the advantages of Traditional Chinese medicine (TCM) in improving the efficacy and reducing the toxicity of radiotherapy.

## 1 Introduction

Radiotherapy is the mainstay of treatment for patients with non-small cell lung cancer (NSCLC), and usually combined with surgery, chemotherapy, immunotherapy, and targeted therapy (1–6). The radiation commonly used in radiotherapy mainly includes photo radiation, such as X-ray and γ-ray and particle rays such as neutrons, electrons, protons and heavy ions (2, 7–10). However, no matter what radiation and dose segmentation methods are used, radioresistance will inevitably occur, leading to radiotherapy failure and local recurrence. Simply increasing the radiation dose does not improve survival benefits but leads to adverse reactions and poor prognosis (11).

Radioresistance of NSCLC can be classified as inherent radioresistance and acquired radioresistance, of which acquired radioresistance plays a major role. Various mechanisms that cause radioresistance run through the whole process of radiotherapy. Whether they cause inherent radioresistance or acquired radioresistance is relative. We have simply classified them according to their leading role in different stages of radiotherapy. Acquired radioresistance is mainly related to DNA damage repair, tumor microenvironment (TME) remodeling by inflammation, immune response, tumor metabolic reprogramming, tumor microbiota and senescence cells, and epithelial-mesenchymal transition (EMT) (12–20). Inherent radioresistance is mainly associated with oncogenic mutation, cancer stem cells (CSCs), and tumor hypoxia (21–29).

Targeted drugs or small molecule drugs combined with radiotherapy are used in the treatment of NSCLC. However, there is no systematic review on the mechanism of radioresistance and radiosensitizers of NSCLC. Therefore, we will discuss the radioresistance mechanisms of NSCLC and the study of drugs that can enhance the efficacy and reduce the toxicity of radiotherapy, especially TCM. So as to provide a theoretical basis for clinical development of anti-tumor drugs in combination with radiotherapy.

## 2 Mechanisms of acquired radioresistance

Acquired radioresistance occurs mainly with radiation induction, the underlying mechanisms involve DNA damage repair and EMT in cancer cell itself, as well as changes in TME such as inflammation, immune response, tumor metabolic reprogramming etc. (15, 16, 19, 30–32). Acquired radioresistance may enhance invasion and metastasis of surviving cancer cells after primary radiotherapy and affect the prognosis and life quality of patients.

### 2.1 DNA damage repair and radioresistance

**Figure 1:**
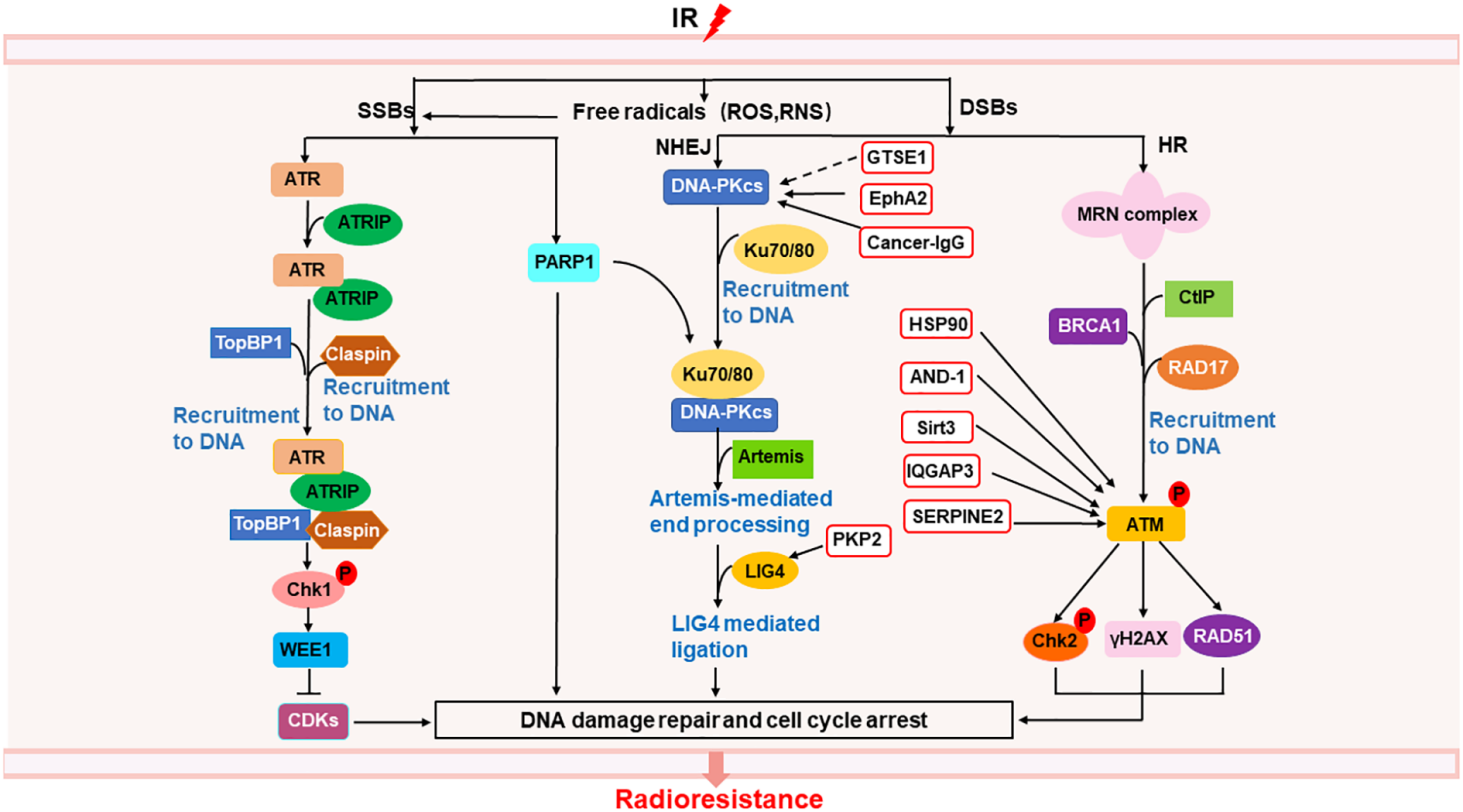
Mechanisms of radioresistance caused by DNA damage repair in NSCLC. Radiation induced DNA damage response can activate the DNA repair pathway, of which SSBs can be repaired through ATR/Chk1 pathway and PARP1, DSBs can be repaired through NHEJ and HR. In the red box are proteins related to the repair of DSBs that may become new targets for radiosensitization. The solid line represents a clear target, and the dotted line represents a possible action through this target.

Radiation causes direct DNA single-strand breaks (SSBs) and double-strand breaks (DSBs), as well as indirect DNA damage from oxidative stress such as ROS and reactive nitrogen species (RNS) (30)(**Figure 1**). SSBs is repaired by poly (ADP-ribose) polymerase (PARP) (33), as well as ATR/Chk1 pathway (34). ATR can be activated by recruitment to its partner protein ATRIP, binding to topoisomerase (DNA) II binding protein 1 (TopBP1) and claspin, phosphorylated Chk1 (34), activated Chk1 phosphorylated WEE1, leading to cell cycle arrest and DNA repair (35), resulting in radioresistance of NSCLC (36, 37). DSBs are repaired by homologous recombination (HR) and non-homologous ending joining (NHEJ) (33). In HR, the MRN complex comprising meiotic recombination 11 homolog1 (MRE11), ATP-binding cassette-ATPase (RAD50), and Nijmegen breakage syndrome protein 1 (NBS1), binds to C-terminal binding protein (CtBP)-interacting protein (CtIP) and BRCA1, was recruited by RAD17 to DSB sites, activated ATM, inducing the phosphorylation Chk2 and H2AX, as well as accumulation of RAD51, thus leading to cell cycle arrest and DNA repair (34, 35, 38, 39). Radiation delayed tumor growth and increased local control in *Atm* deletion tumor model of lung adenocarcinoma (40). In NHEJ, DNA-dependent protein kinase catalytic subunit (DNA-PKcs) is directly recruited to DNA damage sites *via* Ku heterodimers (Ku70/80), inducing Artemis-mediated end processing and DNA ligase 4 (LIG4) mediated ligation, leading to DNA repair (35). Besides, heat shock protein 90 (HSP90) (41), acidic nucleoplasmic DNA binding protein1 (AND-1) (42), sirtuin 3 (Sirt 3) (43), IQ motif containing GTPase-activating protein 3 (IQGAP3) (44), and serine proteinase inhibitor clade E member 2 (SERPINE2) (45) can enhance radioresistance *via* activating ATM to activate the HR pathway in NSCLC. G2 and S phase-expressed 1(GTSE1) (46), plakophilin2 (PKP2) (47), ephrin type-A receptor 2 (EphA2) (48, 49), and cancer-derived IgG (cancer-IgG) (50) can participate in NHEJ pathway, leading to radioresistance in NSCLC. AAA ATPases RUVBL1/2 can activate both HR and NHEJ pathway, inducing radioresistance in NSCLC (51). In addition to Chk1 and Chk2, WEE1 can also regulate cell cycle arrest by inhibiting the phosphorylation of cyclin-dependent kinases (CDKs) (52, 53), inducing radioresistance (54). Thus, targeting DNA repair proteins or cell cycle regulator may overcome radiation-induced radioresistance in NSCLC.

It can be seen from the above that tumor cells have multiple ways to repair radiation-induced DNA damage. Drugs targeting a single target may not have obvious effect on overcoming radioresistance, and are prone to drug resistance. Therefore, the combination of multiple targets for DNA repair will be the direction of tumor treatment in the future, but how to effectively combine the targets still needs more research.

### 2.2 Tumor microenvironment remodeling and radioresistance

**Figure 2:**
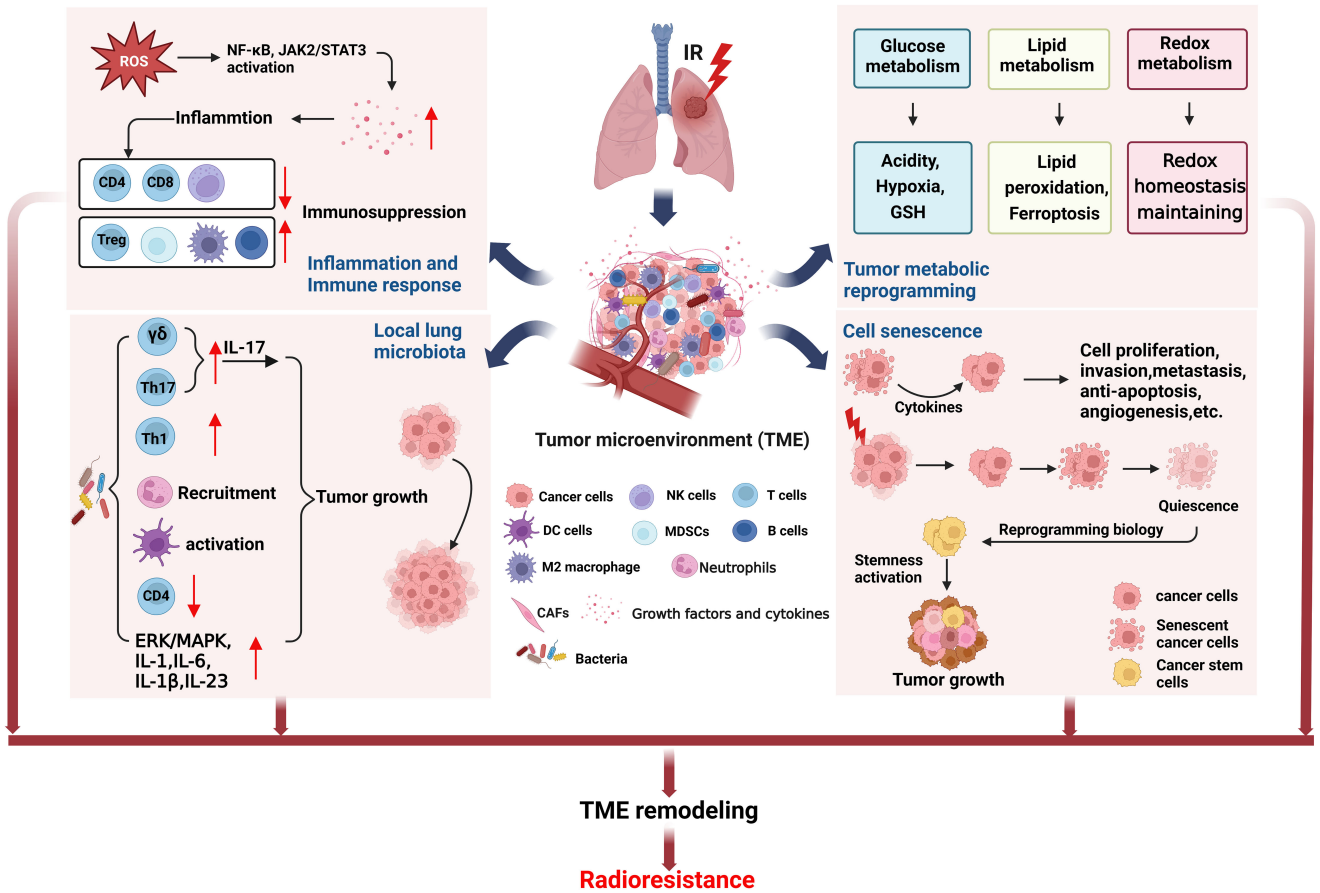
TME remodeling may lead to radioresistance of NSCLC. The TME changed in NSCLC cells surviving after radiotherapy. Radiation induced inflammation and immune response can cause immunosuppression. The local lung microbiota can induce tumor growth by regulating the immune cells. Tumor metabolic reprogramming after radiation will lead to increase of acidity, hypoxia, and GSH production, unbalance of ferroptosis, and redox homeostasis maintaining, being conductive to tumor growth. Cell senescence after radiotherapy can also induce cell proliferation and tumor growth. The above factors together form TME remodeling that promotes tumor growth, metastasis and recurrence after radiotherapy. (This figure is adapted from an image created from BioRender.com).

Tumor microenvironment (TME) is closely related to tumor progression, and the remodeling of TME after radiotherapy can lead to radioresistance (18). Here, we focus on the effects of inflammation and immune response, tumor microbiota, tumor metabolic reprogramming, and cell senescence on TME to clarify the role of radiation-induced TME remodeling in the radioresistance of NSCLC (**Figure 2**).

#### 2.2.1 Inflammation and immune response

Radiation generated ROS and RNS can activate NF-κB, Janus-associated kinase (JAK)2/signal transducer and activator of transcription (STAT)3 pathway to increase the release of growth factors and cytokines, such as VEGF, TGF-β, IL-1, IL-8, TNF-α and IL-6 etc. (15, 31, 55, 56), which are involved in inflammation and immune response (18, 57–60). TGFβ and TNFα in TME are very important in the formation and development of tumor (61). Our previous study found that irradiation of NSCLC cell lines A549 and H1299 can cause overexpression of TGFβ and TNFα, resulting in HIF1α activation and cause genomic instability of BMSCs and may promote tumor progression (62). We also found that TGFβ mainly causes short-term side effects, while TNFα mainly causes long-term side effects. This may provide a guideline for clinical use of combined drugs at different stages of radiotherapy for NSCLC. In addition, radiation-induced inflammation reduces tumor-suppressing immune cells such as CD4+T cells, CD8+T cells, and natural killer (NK) cells, while tumor-promoting immune cells such as regulatory T cells (Treg), myeloid-derived suppressor cells (MDSC), and M2 tumor-associated macrophages (TAMs) increase, eventually forming an immunosuppressive environment, and high level of TGFβ (19, 63). Moreover, M2 macrophages and B cells infiltrate into TME also participate in the radioresistance of NSCLC (64, 65). Thus targeting inflammation not only enhance the tumor killing effects of radiotherapy, but also protect the normal cells and tissues from radiation-induced bystander effects.

#### 2.2.2 Tumor microbiota

It is well known that intestinal microbiome can regulate host physiological and pathological processes through metabolism, inflammation and immune response (66, 67). On the one hand, tumor microbiota can enhance anti-tumor immunity through STING signal activation, T and NK cell activation. On the other hand, it can upregulate ROS, induce anti-inflammatory environment, and inactivate T cell to promote immunosuppression and cancer progression (68). Moreover, tumor microbiota stimulates γδT cells to produce IL-17 that triggering inflammation and cancer progression (69), and increases Th17 cells that enhancing lung cancer proliferation and angiogenesis (70). It is found that the dysbiosis of local lung microbiota in patients with advanced lung cancer can lead to Th1, Th17, γδT cells and PD-1 positive T cells increase, neutrophils recruitment, dendritic cells (DCs) activation, CD4+T cells decrease, cytokines IL-1, IL-1b, IL-6, IL-17, IL-23 upregulation, as well as upregulation of ERK/MAPK and inflammation pathways, all these above alterations promoted tumor progression (71). Although there is no research report on the correlation between tumor microbiota and radioresistance, there is no doubt that tumor microbiota will participate in tumor response to radiotherapy through inflammation and immune response and affect the final efficacy. The microbiome may serve as an indicator for cancer diagnosis or prognostic assessment and could be used as potential targets to develop new cancer therapies.

#### 2.2.3 Tumor metabolic reprogramming

Tumor metabolic reprogramming after radiotherapy, such as glucose metabolism, lipid metabolism, and redox metabolism, contributes to TME remodeling and acquired radioresistance (19). The increased expression of glucose transporter1 (GLUT1), pyruvate kinase M2 isoform (PKM2), and lactate dehydrogenase (LDHA) after irradiation promoted glycolysis (19, 72, 73), while the expression of glycogen synthetase 1 (GYS1) was also up-regulated, leading to glycogen accumulation (74). These changes enable cancer cells to survive, proliferate and resist radiation. In addition, pentose phosphate pathway (PPP) after radiation induced more NADPH production, which promoted the production of reduced glutathione (GSH) (19), may lead to radioresistance of NSCLC.

IR can induce the expression of ACSL4, which is a lipid metabolizing enzyme in cancer cells, to increase lipid peroxidation and ferroptosis (75). Intriguingly, IR can also induce the expression of ferroptosis inhibitors SLC7A11 and GPX4, causing radioresistance (75). Perhaps, radioresistant cells have formed a balance between promoting ferroptosis and inhibiting ferroptosis, enabling cells to survive. In radioresistant lung cancer cells, the expression of lipid Droplet (LD) and ferritin heavy chain (FTH1) increased and correlated with each other, which can be targeted and synergistically inhibit tumor radioresistance (76). In addition, the activation of sPLA2-PKCδ-MAPKs-cPLA2α pathway promoted tumor progression and radioresistance in NSCLC (77).

ROS levels are elevated in lung cancer radioresistant cells, which exhibit radioresistance by maintaining oxidative stress and NRF2-dependent metabolic adaptation (78). The expression of TP53-regulated inhibitor of apoptosis 1 (TRIAP1) in NSCLC cell lines A549 and H460 increased after irradiation, and caused upregulation of antioxidant proteins such as thioredoxin-related transmembrane protein (TMX) 1, TMX2, thioredoxin (TXN), glutaredoxin (GLRX) 2, GLRX3, peroxiredoxin (PRDX) 3, PRDX4 and PRDX6, participated in redox metabolism and enhanced the ROS scavenging, leading to radioresistance in NSCLC (79).

In conclusion, NSCLC cells that survived after radiation have altered metabolism that favors cell proliferation and tumor progression. Therefore, targeting tumor metabolism will be an effective measure to improve the radiotherapy prognosis and survival rate of NSCLC patients.

#### 2.2.4 Cell senescence

Studies have shown that induction of premature senescence can radiosensitize NSCLC cells (80). On the contrary, the cytokines secreted by senescent cells which not killed by radiation will affect the adjacent surviving cancer cells in TME, stimulate tumor cell proliferation, invasion and metastasis, escape apoptosis, induce angiogenesis, and promote tumor phenotype (20). In addition, lung cancer cells survived from radiotherapy experienced senescence and entered a dormant state, reprogrammed their biology months or years after irradiation, reactivated stemness, and produced tumors with enhanced growth and metastasis (81).

To sum up, the TME itself is inflammatory, radiation further promotes the release of inflammatory factors, aggravates inflammatory reaction, causes immunosuppression, and hinders anti-tumor immunity. In addition, tumor microbiota can activate inflammatory pathways to promote tumor progression. Moreover, the oxidative stress reaction induced by radiation promotes the occurrence of tumor metabolism reprogramming, causes the depletion of T cells, and makes tumor cells resistant to radiation. The senescent cells in TME can promote tumor proliferation, invasion and metastasis by secreting cytokines. The interaction of the above factors leads to TME remodeling and radioresistance. In addition to these above, tumor hypoxia also play a key role in TME. It is a challenge to overcome radioresistance by targeting TME, because any single targeting action may not achieve the desired therapeutic effect.

### 2.3 Epithelial-mesenchymal transition and radioresistance

**Figure 3:**
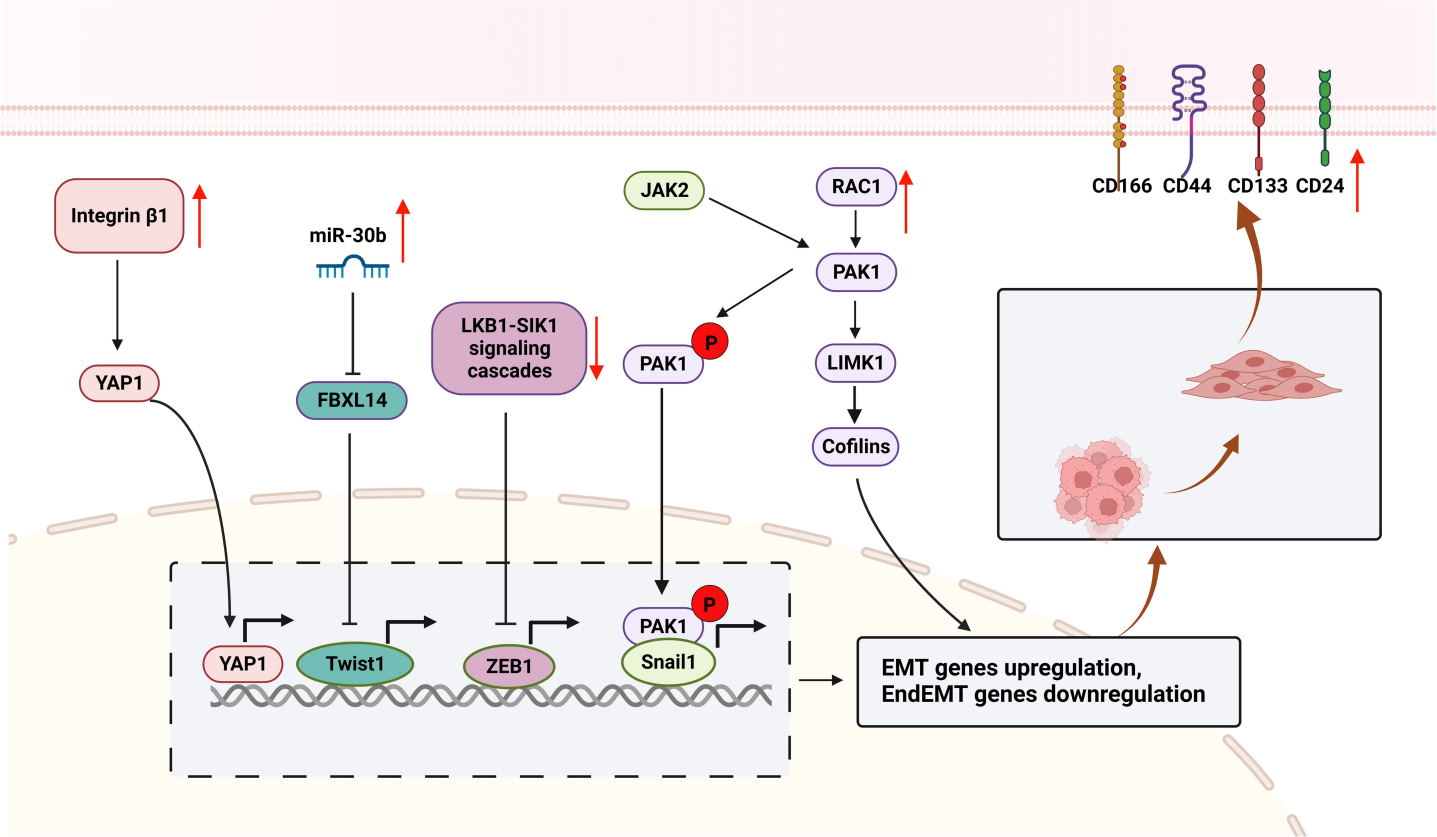
Mechanisms of EMT contributes to radioresistance in NSCLC. The surviving cells included enriched CSCs subpopulations that highly expressed CSCs markers (CD24, CD44, CD133, and CD166). miR-30b was upregulated and targeted to FBXL14, activating the transcription factor Twist1, resulting in increased expression of Vimentin, N-cadherin and Fibronectin, and decreased E-cadherin expression. Integrin β1 is overexpressed after radiotherapy and targets YAP1 to activate ATM/Chk2 signaling, leading to EMT. Furthermore, RAC1 is upregulated in radioresistant NSCLCs, activates the RAC1/PAK1/LIMK1/Cofilins pathway and the PAK1/Snail pathway, causes EMT and promotes tumor metastasis and invasion. LKB1/SIK1 signaling is downregulated after radiotherapy, prompting activation of the transcription factor ZEB1, leading to EMT and tumor metastasis and invasion. (This figure is adapted from an image created from BioRender.com).

Radiotherapy induced EMT and CSCs characteristics, enhanced the invasion and metastasis of cancer cells surviving after primary radiotherapy, leading to acquired resistance of NSCLC, which in turn weakened the radiation-induced tumor killing effect (16, 17, 82). TWIST1 (83, 84) increased after radiation in NSCLC, resulting in downregulation of E-cadherin, overexpression of Snail1, Vimentin, N-cadherin, and PDGFR (85), which promotes cell proliferation, invasion and vascular regeneration, leading to tumor metastasis and radioresistance. PDGF secretion also increased after radiation and resulted in radiation-induced pneumonitis and fibrosis (86), PDGF binds to PDGFR to induce proliferation and vascular regeneration. In addition, Yes-associated protein 1 (YAP1) (87), Snail1 (88), PAK1-LIM domain kinase 1 (LIMK1)-cofilins signaling (89), and zinc-finger E-box-binding homeobox factor 1 (ZEB1) (90)were upregulated after radiotherapy, leading to EMT (**Figure 3**). Radiation survived NSCLC cells also show cancer stemness properties, such as highly expressed CD166, CD24, CD44, Sox-2, CD133, etc. (76, 85, 91). EMT are critical targets and biomarkers for radiotherapy, radiosensitizers targeting EMT are promising treatment for NSCLC in clinical.

## 3 Mechanisms of inherent radioresistance

Inherent radioresistance is an important factor leading to radiotherapy failure, which is mainly related to the gene mutation status of tumor cells, hypoxia status, distribution of CSCs (28, 92, 93), etc.

### 3.1 Mutation status of oncogenes and radioresistance

**Figure 4:**
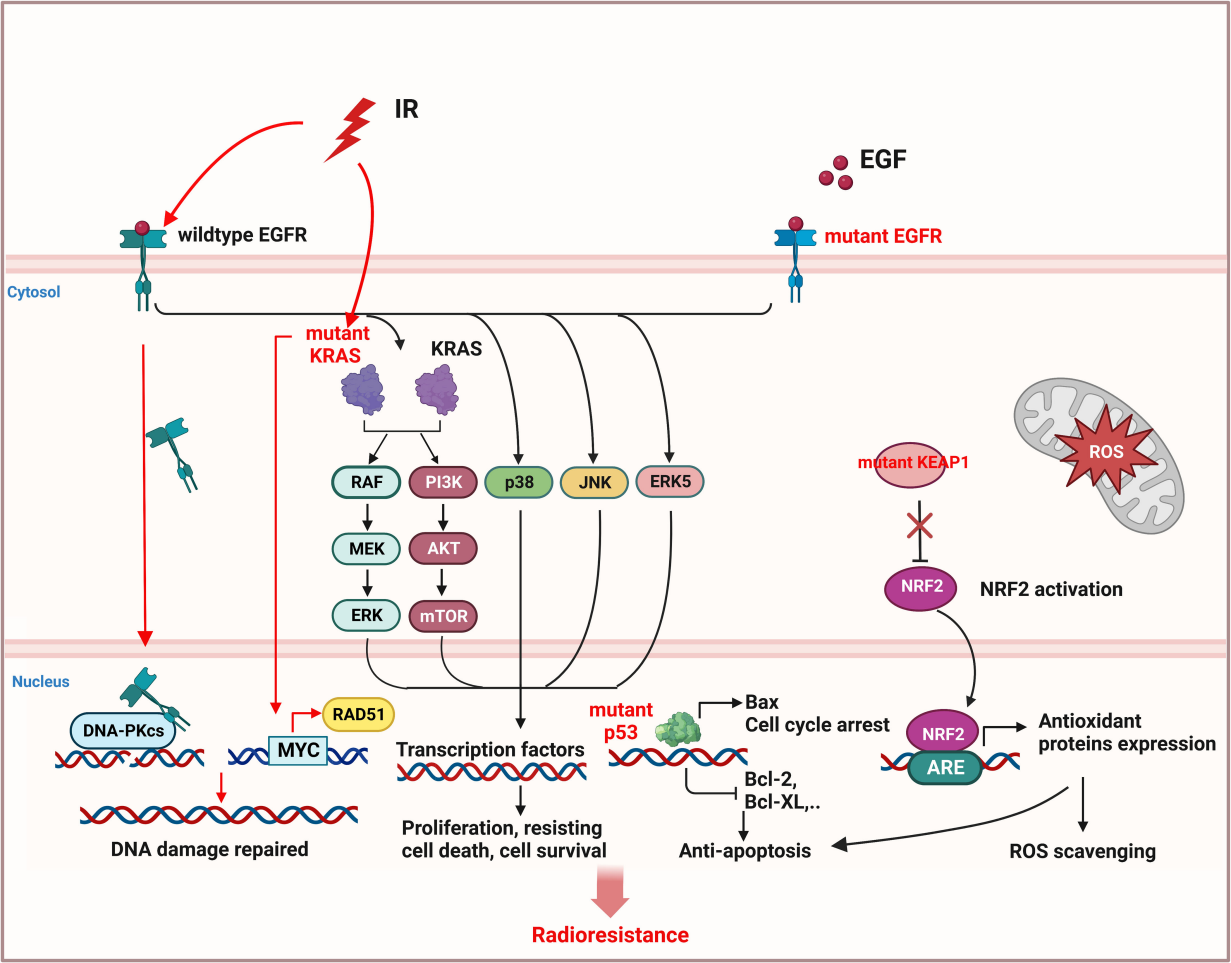
Inherent radioresistance is related to the mutation status of oncogene or tumor suppressor gene in NSCLC. Wildtype EGFR and mutant EGFR both caused cell proliferation and resisting cell death by activation of downstream pathway. In addition, wildtype EGFR can be stimulated by radiation and translocate to nucleus to activate DNA-PKcs, leading to DNA repair. Mutant KRAS can also promote the expression of DNA repair protein RAD51 by activating MYC and improving DNA repair. Mutant p53 will induce anti-apoptosis of NSCLC cells. Mutant KEAP1 cannot inhibit activation of NRF2, leading to the expression of antioxidant proteins to eliminate ROS. (This figure is adapted from an image created from BioRender.com).

Mutations of oncogenes, such as epidermal growth factor receptor (EGFR) (94, 95) and kirsten rat sarcoma viral oncogene (KRAS) (96), or tumor suppressor genes such as Kelch-like ECH-associated protein 1 (KEAP1) (97, 98) and tumor protein P53 (TP53) (99, 100), can cause activation of cell proliferation and resisting cell death signals in NSCLC, leading to radioresistance (**Figure 4**).

EGFR is overexpressed in 40% to 80% of patients with NSCLC and is associated with poor prognosis (101, 102). Radiation can stimulate wild-type EGFR to enter nucleus and bind with DNA-PK catalytic subunit (DNA-PKcs) to promote DNA repair, thus leading to radioresistance of NSCLC (103, 104). However, the NSCLC with mutant EGFR is not necessarily more sensitive to radiation (94), since the mutant EGFR can trigger the downstream pathways, including extracellular signal-regulated kinase (RAS/ERK), p38MAPK, c-Jun N-terminal kinase (JNK), and ERK5, and also activate PI3K/AKT/mTOR signaling pathway, leading to cell proliferation and anti-apoptosis, migration, DNA repair, cell cycle arrest, etc., which in turn increases the radioresistance of NSCLC (22, 23, 103, 105–108).

KRAS can regulate cell growth, differentiation and apoptosis, its mutations mainly occur at codons 12 and 13, and have been found approximately in 20-30% of NSCLC tumor samples (109). KRAS-mutant NSCLC cells are more radioresistant than KRAS wild-type cells (96, 110) or EGFR-mutant cells (21), because mutant KRAS can upregulate RAD51 expression through oncogene MYC, thereby enhancing DNA damage repair and cell survival (111). In addition, EGFR-dependent chromatin condensation, namely mitotic-like concentrated chromatin (MLCC), can protect KRAS-mutant NSCLC cells from ionizing radiation (IR)-induced DSBs and premature senescence (110), and enhance the expression of CSC marker protein CD133 *via* osteopontin/EGFR pathway, promoting tumor invasion and radioresistance (112).

KEAP1/nuclear factor E2-related factor (NRF2) mutation happens approximately 30% of NSCLC (92), and 7% of patients have co-mutations of KEAP1/NRF2 and EGFR (113). Mutant KEAP1 lost its inhibitory effect on NRF2 in cytoplasm, the activated NRF2 translocated to the nucleus and activated the expression of antioxidant proteins by binding to antioxidant-responsive elements (AREs), resulting in the scavenging of ROS and anti-apoptosis of tumor cells, leading to unlimited proliferation, metastasis and radioresistance of NSCLC (114–116).

TP53 mutation occurs in more than half of human cancers, including NSCLC (92). p53 can not only induce cell cycle arrest, senescence, and apoptosis, but also regulate tumor metabolism, promote ferroptosis, and inhibit tumor development (117). Mutant p53 can neither induce the expression of pro-apoptotic protein Bax, nor inhibit the expression of anti-apoptotic proteins (Bcl-2, Bcl-xl) (118), which makes cancer cells survive in radiotherapy (119). Compared with single mutations in TP53, NSCLC with co-mutation in KRAS and TP53, or co-mutation in TP53 and KEAP1 are more resistance to IR-induced cell death (100, 114).

In addition to the oncogenic mutation, high levels of inhibitor of apoptosis proteins, such as cIAP1/2, XIAP, and survivin, participate in cell death and survival by binding and inhibiting caspases, leading to radioresistance of NSCLC (120–123). In addition, microRNAs can also affect the radiosensitivity of NSCLC by regulating the cell proliferation and apoptosis. miR-99a and miR-770-5p can induce apoptosis and improve radiosensitivity (124, 125). On the contrary, miR-410 and miR-208a promote radioresistance by inducing EMT and increasing cell proliferation respectively in NSCLC (126, 127).

According to the potential mechanism of inherent radioresistance mentioned above, genome sequencing can be carried out before treatment to determine the mutation type and formulate effective treatment strategies, so as to improve the tumor-killing rate and reduce the tumor recurrence rate after primary radiotherapy of NSCLC.

### 3.2 Cancer stem cells and radioresistance

**Figure 5:**
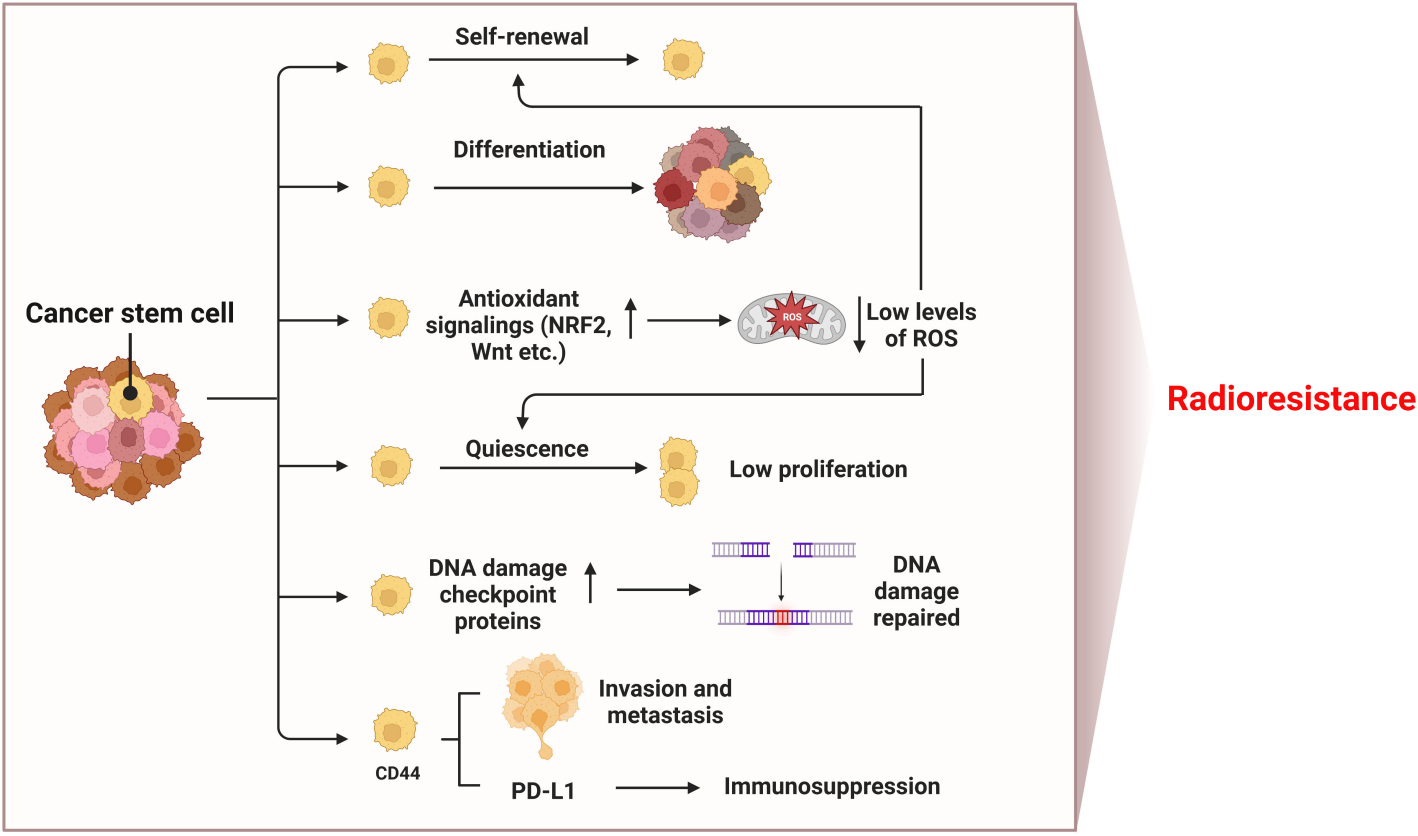
Mechanisms of radioresistance caused by CSCs in NSCLC. Hallmarks of CSCs such as self-renewal and differentiation, low levels of ROS, quiescence, DNA damage repair, invasion and metastasis ability, and immunosuppression induced by upregulated PD-L1 all contribute to radioresistance of NSCLC. (This figure is adapted from an image created from BioRender.com).

Cancer stem cells (CSCs) are subpopulations of tumor cells with the ability to self-renew and differentiate into heterogeneous tumor cells, promote tumor growth and metastasis through unlimited proliferation and migration, leading to tumor recurrence and treatment failure (128–131). Antioxidant signaling pathways (NRF2, Wnt, etc.) in CSCs are activated, and more antioxidant proteins are expressed to clear ROS, so as to maintain a low level of ROS in cells, which contributes to the quiescence and self-renewal of CSCs (130, 132–134) (**Figure 5**). In addition, CSCs have a strong ability to repair DNA damage (135). CD133 is one of the markers of CSCs. As early as 2006, Bao et al. reported that CD133-positive glioma cells showed higher expression of DNA damage checkpoint proteins (i.e. ATM, Rad17, Chk1 and Chk2) than CD133-negative cells (136). In 2014, Desai et al. clarified that CD133-positive NSCLC cells highly express DNA damage repair proteins RAD51 and Exo1, which can promote radioresistance, but had cell type specificity (137). Therefore, low levels of ROS and enhanced DNA damage repair make CSCs insensitive to radiotherapy, leading to tumor relapse (138). CD44 is another CSCs marker, which is related to tumor recurrence and metastasis, as well as prediction of the outcome after radiotherapy (139, 140). The overexpression of CD44 promotes the proliferation of NSCLC, and up-regulates the expression of PD-L1 to promote tumorigenesis, immunosuppression and chemotherapy resistance (141, 142). It is worth noting that due to the low sensitivity of CSCs to IR, the CSCs not killed by radiation will be enriched, which leads to acquired radioresistance. Therefore, the treatment efficiency of NSCLC can be improved by inducing CSCs to differentiate to reduce their proportion in tumor tissues, or by combining drugs targeting key proteins of CSCs antioxidant pathways or surface marker proteins with radiotherapy.

### 3.3 Tumor hypoxia and radioresistance

**Figure 6:**
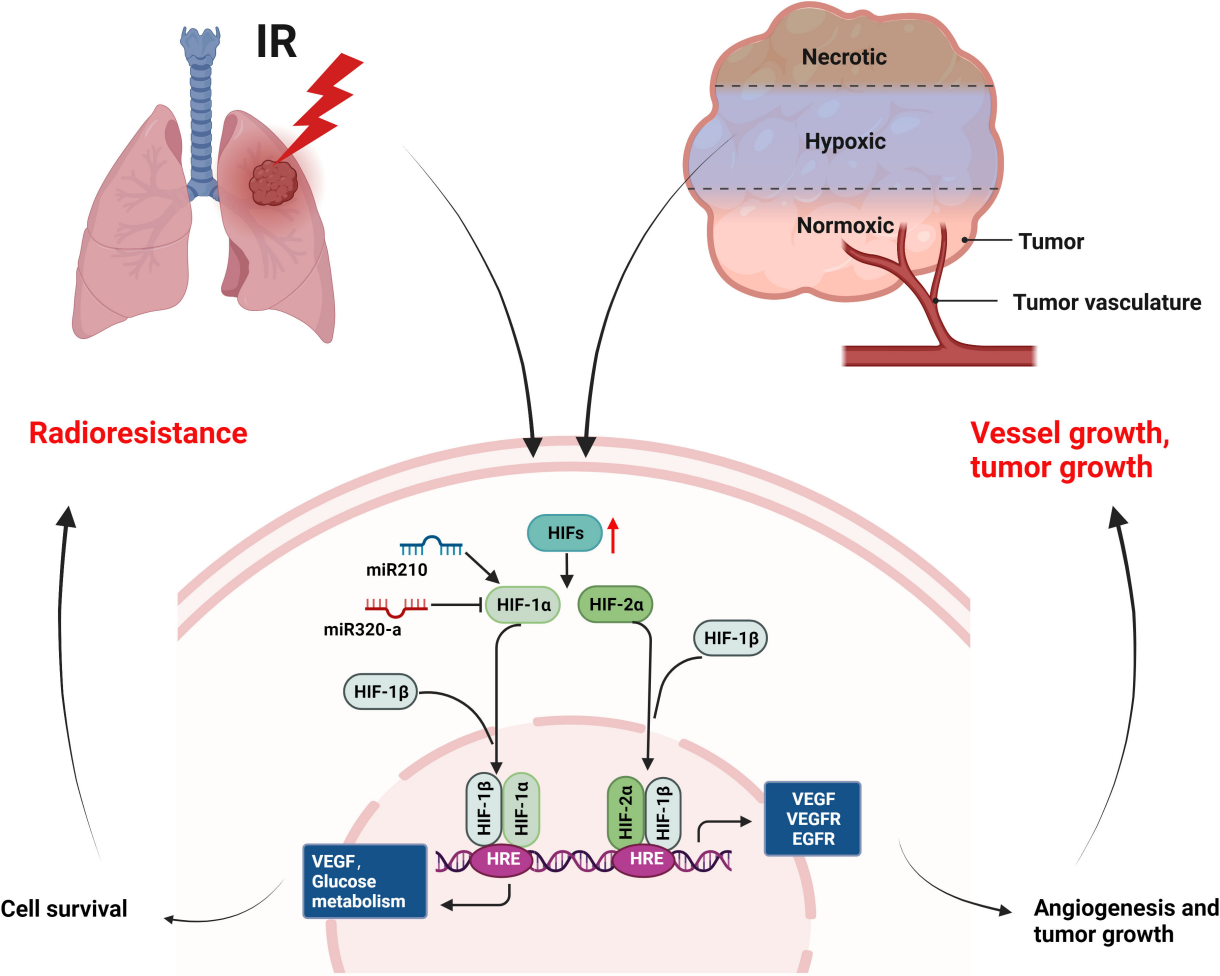
Mechanisms of radioresistance caused by hypoxia in NSCLC. Hypoxia environment of tumor and radiation can activate the expression of HIFs, which will induce the expression of growth factors (VEGF, VEGFR, EGFR etc.) and improved glucose metabolism, leading to cell survival, angiogenesis, and tumor growth of NSCLC. (This figure is adapted from an image created from BioRender.com).

As one of the cancer hallmarks, hypoxia induces angiogenesis, tumor invasion, metastasis, and treatment resistance (27, 28). Hypoxic tumor cells are very insensitive to radiation (143). In this article, we mainly introduce the research on HIFs and radioresistance in NSCLC (**Figure 6**). Hypoxia environment and radiation activate HIFs (144, 145), activated HIF-1α and its partner subunits HIF-1β enter nucleus and bind to hypoxia-responsive element (HRE) to trigger gene transcription regulating VEGF and glucose metabolism etc., leading to cell survival (146–148). HIF-2α mainly induces angiogenic factors, such as VEGF, VEGFR, EGFR etc., promoting angiogenesis and tumor growth (149–151). Research showed that the upregulation of HIF-1α enhanced the radioresistance of NSCLC (152). Conversely, the decrease of HIF-1α in acidic environment caused radioresistance in NSCLC cells and blocking simultaneously of both HIF-1α and HIF-2α radiosensitized NSCLC, suggesting the role of HIF-2α in radioresistance (153). In addition, miR-210 promoted the hypoxic phenotype of NSCLC cells and increased radioresistance by inducing and stabilizing HIF-1α (154). By contrast, another microRNA, miR-320a targeted HIF-1α to promote methylation of PTEN, thereby reducing the radioresistance of NSCLC *in vitro* and *in vivo* (155). In conclusion, targeting HIFs may enhance radiosensitivity of NSCLC in clinical, but the pH value should be considered when targeting HIF-1α.

## 4 Radiosensitizing non-small cell lung cancer

As we discussed above, the mechanisms of radioresistance of NSCLC are complex and not isolated from each other. Whether surgery, chemotherapy, targeted therapy, or immunotherapy, the goal of their combination with radiotherapy is to kill tumor cells, but what cannot be avoided is limited effect, tumor recurrence and metastasis, as well as side effects.

At present, chemotherapy drugs, targeted drugs, immune checkpoint inhibitors, etc. can be used as radiosensizitizers in NSCLC, but these drugs are prone to drug resistance and toxic side effects (11, 30, 156, 157). In addition, most of the current studies about radiosensitizers used in NSCLC is still limited to experimental research, and only a few drugs have been used in clinical. We summarized the drugs or agents for NSCLC radiosensitization in **Table 1**, including antibody drugs and small molecule inhibitors from non-natural sources. In addition to drug toxicity, radiotherapy itself can also cause lung toxicity in patients, manifesting as different grades of pneumonia, pulmonary fibrosis (32, 183). In addition, the mechanisms of radioresistance are complex, and the efficacy of these single targeted drugs is limited. Therefore, it is an urgent need to develop drugs targeting multiple targets, with synergistic effects and low toxicity in NSCLC radiotherapy.

**Table 1:** Non-natural products radiosensitize NSCLC by targeting key proteins.

Mechanism of action	Targets	Radiosensitizing modulators	Experimental models/(*in vitro* or *in vivo* or clinical)	Conclusion	Reference
Targeting DNA repair	PARP	Olaparib+ML216	*In vitro* and *in vivo*	PARP inhibitors and BLM helicase inhibitors synergistically radiosensitized NSCLC.	(158)
	PARP	niraparib	*In vitro* and *in vivo*	Niraparib activated anti-tumor CD8+T cells in the tumor microenvironment through the STING/TBK1/IRF3 pathway in EGFR-mutant NSCLC.	(159)
	ATM	KU55933	*In vitro*	ATM inhibitors radiosensitized NSCLC by inhibiting IR-induced EGFR activation and can be used in EGFR-resistant tumor cells for radiotherapy.	(160)
	DNA-PK	AZD7648	*In vitro* and *in vivo*	DNA-PK inhibitors are promising radiosensitizers for treatment of NSCLC.	(161)
	Telomerase	BIBR1532	*In vitro* and *in vivo*	Inhibiting telomerase function can radiosensitize NSCLC.	(162)
	AND-1	CH-3 compound	*In vitro* and *in vivo*	Inhibition of AND-1 will be a promising strategy for enhancing radiosensitivity in NSCLC.	(42)
	RUVBL1/2	Compound B	*In vitro* and *in vivo*	RUVBL1/2 inhibition combined with radiotherapy may be effective treatment for NSCLC.	(51)
Targeting cell cycle	BET	JQ1	*In vitro* and *in vivo*	BET inhibition may be a potential treatment for radiosensitizing NSCLC.	(163)
	CDK4/6	Palbociclib/	*In vitro*	Combining inhibition of CDK4/6 and radiotherapy will be promising strategy for patients with p53-wild type NSCLC.	(164)
	CDK4/6	Abemaciclib	*In vitro* and *in vivo*	Abemacilcib combined with radiotherapy radiosensitized NSCLC in preclinical models.	(165)
Targeting inflammation	IKKβ	IMD03544	*In vitro* and/or *in vivo*	Blockade of IKK-NF-κB signaling will be benefit for radiotherapy in NSCLC.	(60, 166)
	TGF-β	LY364947	*In vitro* and *in vivo*	TGF-β inhibition combined with radiotherapy enhanced therapeutic efficacy in NSCLC.	(167)
	STAT3	Niclosamide	*In vitro* and *in vivo*	Inhibition of STAT3 signaling will be effective treatment for NSCLC to radiotherapy.	(59)
	JAK2	TG101209	*In vitro* and *in vivo*	Targeting JAK2 may enhance radiotherapy response in KRAS-mutant NSCLC.	(168)
Targeting immune suppression	PD-L1	anti-PD-L1 antibody	*In vitro* and *in vivo*	Anti-PD-L1 antibody combined with radiotherapy synergistically enhanced anti-tumor immunity in NSCLC.	(169)
	PD-1	Nivolumab	Clinical phase II (Enrollment: 65)	No published results to date	(170)
	PD-1	pembrolizumab	Clinical phase I/II (Enrollment: 104)	Pembrolizumab combined with radiotherapy may be effective in certain subgroups of NSCLC patients with low PD-L1 expression	(171)
	CTLA-4	Ipilimumab	Clinical phase I/II (Enrollment: 39)	CTLA-4 blockade and radiotherapy synergistically induced robust antitumor T cell responses	(172)
Targeting glutamine metabolism	Glutaminase	CB-839	Clinical phase I/II (Enrollment: unknown)	KEAP1/NFE2L2 mutations is associated with radioresistance and local recurrence, and glutaminase inhibition enhanced radiotherapeutic effects in NSCLC.	(97)
Targeting cell proliferation	EGFR	Erlotinib, gefitinib	Clinical phase II (Enrollment: 10)	EGFR-TKI combined with radiotherapy achieves long-term control of stage IV NSCLC with EGFR mutations.	(173)
	EGFR/HER2/HER3	Pan-HER	*In vitro* and *in vivo*	Pan-HER exerted anti-proliferation and tumor growth-delay effect by inhibiting multiple HER members to enhance radiation response in NSCLC.	(174)
	ERK5	XMD8-92/ERK5 knockdown	*In vitro* and *in vivo*	ERK5 contributes to lung cancer development and radioresistance and can be a novel target for radiotherapy in NSCLC.	(23)
	EGFR and Norch	Anti-EGFR/Notch CrossMab (CT16)	*In vitro* and *in vivo*	Combined blocking of EGFR and Notch signaling enhanced radiation response in NSCLC.	(175)
	EGFR and COX2	Afatinib+celecoxib	*In vitro*	Combined inhibition of EGFR and COX-2 radiosensitized NSCLC.	(176)
Targeting cell apoptosis	mTOR	miR-99a	*In vitro* and *in vivo*	MiR-99a enhanced radiosensitivity *via* targeting mTOR in NSCLC.	(124)
	miR-410		*In vitro* and *in vivo*	MiR-410 increased radioresistance *via* targeting PTEN/PI3K/mTOR pathway in NSCLC.	(126)
	Bcl-XL	BXI-61, BXI-72	*In vitro* and *in vivo*	Small molecules targeting Bcl-XL conquered acquired radioresistance in NSCLC.	(177)
	Anti-apoptotic Bcl-2 family	ABT-737	*In vitro* and *in vivo*	ABT-737 radiosensitized K-ras mutant NSCLC by inhibiting the activity of Bcl-2 family proteins.	(178)
	Bak	BKA-073	*In vitro* and *in vivo*	Bak activators may be promising anticancer drugs in NSCLC treatment.	(179)
	Bcl-2/Bcl-xL	ABT-263	*In vitro* and *in vivo*	Inhibiting anti-apoptotic Bcl-2 family proteins may enhance radiosensitivity by reducing hypoxia-mediated anti-apoptosis in NSCLC.	(180)
	cIAP1/2	Birinapant	*In vitro*	cIAP1/2 will be promising targets for enhancing radiosensitivity of NSCLC.	(123)
Targeting angiogenesis	VEGFR2	Apatinib	*In vivo*	Apatinib can radiosensitize lung cancer through vascular normalization and hypoxia reduction in the xenograft model	(181)
	VEGF	Endostar	Clinical phage II (Enrollment: 67)	Continuous intravenous endurance combined with concurrent etoposide and radiation prolongs OS in patients with unresectable stage III NSCLC.	(182)

## 5 Application and advantages of TCM in radiotherapy for NSCLC

As an auxiliary means of radiotherapy for NSCLC, TCM can increase the apoptosis of cancer cells, inhibit tumor metastasis, enhance the anti-tumor immunity of patients, regulate the TME homeostasis, thus improving the efficacy of radiotherapy and reducing the recurrence rate (184, 185), which reflecting the basic principles of treating tumor by reinforcing healthy qi to eliminate pathogenic factors of TCM. Some single herbs are frequently used in the treatment of NSCLC (186). Commonly used TCM for reinforcing healthy qi to eliminate pathogenic factors include Ginseng, Codonopsis pilosula, Astragalus, Angelica, and Polygonatum, etc. Astraglus has immunoregulatory, antioxidant, anti-inflammatory, and anti-cancer activities and combined with chemotherapy to increase the efficacy and reduce the toxicity in patients with advanced NSCLC (187, 188). Our previous research found that Astragalus polysaccharides can reduce the radiation induced bystander effects (RIBE) of bone marrow mesenchymal stem cells (BMSCs) caused by X-rays and heavy ions, and reduce the toxicity of normal cells and tissues in lung cancer radiotherapy (189–191). In addition, our research also found that Guiqi Baizhu Decoction, which composed of Astragalus, Angelica, Atractylodes, Paeonia lactiflora, Tangerine peel, Rhubarb, and Licorice, can significantly reduce the radiation inflammatory reaction and immune damage, and prevent intestinal microbial imbalance and metabolic disorders caused by radiation (192). We further found that Guiqi Baizhu Decoction can regulate HIF-1α, AQP4 and Na+/K+-ATPase to reduce hypoxia and oxidative stress, thus treating radiation-induced intestinal edema (193). Moreover, in the study of gastric cancer, we used chemical informatics and cell experiments to verify that Guiqi Baizhu decoction plays a dual role of anti-tumor and immunoregulation by targeting HER2 and PD-L1 through its active components quercetin and isorhamnetin respectively, reflecting the mechanism of multi-point and synergistic effect of TCM in tumor treatment (194).

In recent decades, the advantages of natural products in anti-tumor have become more and more obvious, such as paclitaxel (195) and vinorelbine (196) has been used in clinical for decades. Here we listed the application of TCM compound prescription, single herb, herbal extracts, and small molecules from TCM in the preclinical and clinical research of radiotherapy toxicity reduction and efficiency enhancement for NSCLC (**Table 2**). The various factors that cause radioresistance are not independent, nor only appear in a certain period of time. Moreover, at different stages of radiotherapy, the mechanism that dominates radioresistance is variable and complex. However, TCM can adjust the radiation anti-tumor effect in different ways with the advantage of multi-component and multi-target, improve the efficacy of radiotherapy, reduce the toxicity of radiotherapy and improve the quality of life of patients.

**Table 2:** Natural compound/traditional Chinese medicine used in radiotherapy of NSCLC.

Natural compound/Traditional Chinese medicine	Type of drug	Experimental models /(*in vitro* or *in vivo* or clinical)	Function	Reference
Aidi injection	Compound prescription, composed of extracts from Mylabri, Ginseng Astragalus, and Acanthopanacis senticosi.	Clinical	Heat-clearing and toxin-removing, eliminating stasis and resolve masses. Aidi injection improved PSF and immune function in lung cancer patients and alleviated the myelosuppression, radiation pneumonitis, radiation esophagitis.	(197, 198)
Shengqi Fuzheng injection	Compound prescription, composed of extracts from Codonopsis and Astragalus.	Clinical	Supplement and tonify lung qi. Reduced the inflammatory response and improved immune function by regulating the expression of TNF-α and TGF-β.	(199)
Compound kushen injection	Compound prescription, composed of extracts from Sophorae and Rhizoma Smilacis Glabrae.	Clinical	Clear heat and drain dampness, resolve masses and alleviate pain. Improved efficacy and reduced radiation pneumonitis, radiation esophagitis, and myelosuppression.	(200)
Kangai injection	Compound prescription, composed of extracts from Astragalus, Ginseng, and Oxymatrine.	*In vitro*	Increased FOXO3a-mediated apoptosis and autophagic cell death in cisplatin-resistance A549 cells.	(201)
*Astragalus*	Monomer herb	Meta-analysis	Reduced the toxicity and increased the efficacy of radiotherapy.	(202)
Elemene injection	Herbal extractions from Curcuma	Meta-analysis	Reduce adverse reactions, relieve symptoms.	(203)
*Ganoderma lucidum* polysaccharide	Herbal extractions from Ganoderm	*In vitro* and *in vivo*	Decreased the tumor immune suppression.	(204)
Kanglaite injection (KLTi)	Herbal extractions from Coix agrestis Lour	Clinical	KLTi in combination with radiotherapy improved patient survival but can induce adverse effects such as leukopenia, nausea and vomiting.	(205)
*Astragalus* polysaccharide	Herbal extractions from Astragalus.	*In vitro*	Reduced the radiation-induced bystander effects through regulation of MAPK/NF-κB signaling pathway, or TGF-βR/MAPK/ROS pathway.	(189–191)
Micheliolide	Small molecule derived from Michelia.	*In vitro*	Micheliolide enhanced radiosensitivity *via* inducing the ubiquitination degradation of HIF-1α in p53-null NSCLC.	(206)
Quercetin	Small molecule, derived from a variety of TCM such as Bupleurum, Hypericum, Astragalus, etc.	*In vitro*	Inhibited WEE1 signaling	(54)
Daurinol	Small molecule, derived from Haplophyllum.	*In vitro*	Suppressed serine/threonine family of aurora kinases A/B(AURKA/AURKB) can radiosensitize NSCLC.	(207)
Isorhamnetin	Small molecule, derived from a variety of TCM such as Folium Ginkgo, Astragalus, etc.	*In vitro*	Upregulated IL-13, suppressed NF-κB signaling and increased cell apoptosis.	(208)
Cucurbitacin I	Small molecule, mainly distributed in Cucurbitaceae.	*In vitro* and *in vivo*	Inhibition of STAT3 signaling in CD133- positive NSCLC may enhance radiotherapy effect.	(209)
Phoyunnanin E	Small molecule, isolated from Dendrobium venustum.	*In vitro*	Induce lung cancer cell apoptosis *via* inhibiting survivin.	(210)
Celastrol	Small molecule, derived from Celeastrus.	*In vitro*	Celastrol radiosensitized NSCLC by suppressing the ATP-binding activity of Hsp90.	(211)
Curcumin	Small molecule, isolated from *Curcuma longa* L.	*In vitro* and *in vivo*	Increasing cell apoptosis *via* activating the p53-miR-192-5p/215-XIAP pathway.	(212)
Scutellarin	Small molecule, isolated from Scutellaria altissima L. or S.baicalensis Georgi.	*In vitro* and *in vivo*	Promoted cell apoptosis *via* down-regulating AKT/mTOR pathway.	(213)

## 6 Conclusions

In conclusion, the radioresistance mechanisms of NSCLC are complex. Therefore, prior to radiotherapy gene sequencing should be performed for NSCLC patients to determine the type of mutation, or abnormally expressed genes, proteins, and signaling pathways, thus to develop more effective treatment strategies. In the early stage of radiotherapy, it is suggested to combine EGFR, KRAS and other mutant targeted drugs, drugs to improve DNA damage, immune checkpoint drugs or drugs to target hypoxia. In the middle and late stage of radiotherapy, it is suggested that the combination of drugs targeting EMT or regulating TME can improve the curative effect. TCM can run through the whole radiotherapy process. However, the specific mechanism of TCM in the sensitization of NSCLC radiotherapy needs to be further explored. The combination of effective small molecules extracted from TCM and radiotherapy may provide a new application prospect for the future clinical treatment of NSCLC.

## Author contributions

TZ is responsible for literature review and article writing. LZ and JH are responsible for revising articles. ZM, YYL, YZ, and ZL are responsible for collecting and sorting materials. SZ, YC, and GZ are responsible for sorting charts. YQL provides ideas and guidance for the article. All authors contributed to the article and approved the submitted version.
